# Application of Artificial Intelligence System Based on Wireless Sensor Network in Enterprise Management

**DOI:** 10.1155/2022/2169521

**Published:** 2022-05-27

**Authors:** Kefeng Li

**Affiliations:** Business School, Anyang Normal University, Anyang, Henan 455002, China

## Abstract

With the improvement of the ability to acquire natural information, wireless sensor networks also need to transmit corresponding information in terms of collecting information. Wireless sensor nodes have great application prospects as a key component of wireless sensors. Therefore, different wireless sensors play an important decisive role in the operation of wireless network applications. With the continuous development of wireless sensor networks, existing wireless sensor network nodes exhibit limitations and shortcomings such as inflexible structure, low variability, and low versatility. Specifically, the learning and neural networks obtained by different artificial intelligence expert systems in computing technology are different. On the one hand, it can meet the needs of users for information systems to a certain extent, and on the other hand, it can also help accelerate the development of computer science. At present, the new generation of information technology industry is listed in the seven emerging strategic industries of the country. The new cloud computing technology has gradually expanded to important corporate governance capabilities in terms of information technology. The intelligent application of cloud computing technology replaces traditional enterprise management technology. Efficiency management and risk management can improve the quality and business capabilities of the entire enterprise, improve system applications according to the actual situation of the enterprise, improve system applications, and implement health and the sustainable development of the enterprise, thereby promoting the sustainable development of the computer technology industry.

## 1. Introduction

The wireless sensor is mainly composed of a network system connected by house types. At present, it is mainly a frontier field involving a wide range of disciplines at home and abroad and a relatively high research enthusiasm [1]. The most advanced network technology in the world is embedded computer information. Processing technology can realize the identification of various monitoring through different sensors, as well as the collection of information and data, and then wirelessly transmit it to the hands of each user [2]. Different wireless sensor networks have different technical forms. For example, some organizations are spontaneous, fault-tolerant, fast, and have properties such as small size [3]. The prospects for wireless sensors in urban management and military applications are also very broad [4]. In recent years, with the rapid development of information technology, different technical scopes have made great contributions to transportation, education, medical finance, and other aspects. Information technology has become a major change in production and life [5]. People's lifestyles and work styles will change accordingly. Informatization has also played an important role in the development of China's industrialization and industrialization [6]. The Chinese government has always advocated the integration of the two [7]. In the development of the 17th Party's work, it should promote the rapid development of industry, as well as the advancement of informatization, fiscalization, and national resources [8]. To issue opinions on accelerating the in-depth integration of informatization, it also vigorously promotes the in-depth integration of informatization. Different corporate information plays different roles in the components of corporate strategy [9]. In mobilizing enthusiasm, new synergy needs to be formed, and some corporate information services should also be mentioned to encourage small businesses to use information technology [10]. The ideal cost control should include planning, accounting, monitoring, and evaluation [11]. Traditional corporate cost management models often focus on cost control, using technology and some content that can ignore operating costs to control modern corporate management and gradually establish a mature concept of cost-effectiveness [12]. From the traditional concept of simply focusing on saving costs, it has gradually realized the importance of investment in product development [13]. In addition, dynamically configurable resources can significantly reduce business costs related to purchasing and upgrading software and hardware [14]. Enterprise performance management is the study of how to improve organizational management methods and processes under specific investment and technical conditions so that specific investments can bring the most satisfactory use to the enterprise [15].

## 2. Related Work

According to the literature, it is necessary to use modeling to realize human intelligence in the manufacturing of robots [16]. The concept of artificial intelligence is not a completely new concept. Models and research and development have been explored and expanded. As we all know, human consciousness and thinking processes need to simulate the essence of intelligence and produce smart devices that can react in the same way as human intelligence [17]. The equipment produced can perform image recognition and language recognition processing like a robot, and its thinking ability can also be the same as that of humans [18]. At the same time, the wireless sensor network shown in this article is based on a facility and an organizational network [19]. Different network sensor nodes are divided into different monitoring areas and are also classified according to the nodes where their functions are converged to form the nodes of the sensor network [20]. The literature also pointed out that, in the detection process under the engineering process, the sensor nodes are arranged randomly, and different collected sensor information is sent to the synchronized node in a hopping manner and then sent to the interconnection or satellite, before being sent to the control node at a fixed point [21]. According to the literature, the workflow of wireless sensor network nodes shows that the performance and practicability of nodes are related to the efficiency and operating costs of the entire network. Therefore, high-performance and low-cost nodes are the key to designing wireless sensor networks [22]. In addition, the literature also proposes to analyze the impact of changes in the external environment on the development of China's cloud computing industry from the perspective of politics, economy, society, and technology and analyze the advantages of cloud computing technology over traditional enterprise management information technology and the impact of cloud computing technology on enterprise development. The literature also provides many research perspectives on products and solutions, combined with specific information technology implementation levels, so that cloud computing services can provide some efficient management, reduce management risks, and provide enterprises with efficient cloud computing services. The literature first analyzes the definition, classification, characteristics and other aspects of cloud computing, which will help advance the systematic and complete research on cloud computing theory and clarify the relationship between cloud computing technology and enterprise management and interoperability. In addition, the improvement of efficiency management, risk management and quality of different enterprises, the comprehensive competition of enterprises to enhance the sound and sustainable development of enterprises, and the implementation of sustainable cloud computing are all helpful to promote the development of the industry. In the end, the following conclusion is reached: when implementing enterprise information management, companies should know how to use cloud computing technology wisely, instead of just focusing on traditional information management technology to bring the greatest benefits to cloud computing services.

## 3. Wireless Sensor Network Algorithm and Artificial Intelligence System

### 3.1. Wireless Sensor Network Algorithm

#### 3.1.1. Least Squares Method

The least square method is developed based on the three-way measurement method.

Formula (1) can be obtained from the point-to-point distance equation:

(1) $$\begin{array}{c} {\left(x_{n}-x\right)}^{2}+{\left(y_{n}-y\right)}^{2}=d_{n}^{2}. \end{array}$$

If the nodes of the beacon are the same and are close to each other relative to the node under test, the azimuth error of each node of the beacon can be considered equal, and then the position of the node under test is evaluated according to the following:

(2) $$\begin{array}{c} \hat{x}=\text{argmin}\frac{1}{2}\sum_{i=1}^{n}\frac{{\left(\theta_{i}\left(\hat{x}\right)-\propto_{i}\right)}^{2}}{\delta_{i}^{2}}. \end{array}$$

#### 3.1.2. Positioning Based on RSSI

Based on the RSSI positioning principle, there is a certain functional relationship between the strength of the RSSI signal and the distance during signal propagation. The corresponding rule of this function is that the signal decreases as the distance increases. This function can be used to obtain the relationship between the RSSI value of the signal power value received by the node under test and the propagation distance. The propagation mode is usually specified according to the signal power value.

Consider the following:

(3) $$\begin{array}{c} P_{L}\left(d\right)=P_{L}\left(d_{0}\right)+10n\mathrm{lg}\left(\frac{d}{d_{0}}\right)+X_{0}. \end{array}$$

Use some wireless signals for the same type of propagation, and then, reach the test time at different nodes, using the principle that the position of the node is different from the signal time. This test method is also called the hyperbolic positioning method; because of the arrival time, the difference determines the hyperbola centered on the beacon node. Therefore, the following can be obtained:

(4) $$\begin{array}{c} d_{n}^{2}={\left(x_{n}-x_{0}\right)}^{2}+{\left(y_{n}-y_{0}\right)}^{2}. \end{array}$$

Formula (4) can also be converted as follows:

(5) $$\begin{array}{c} \left(d_{1}+\Delta d_{n}\right)={\left(x_{n}-x_{0}\right)}^{2}{\left(y_{n}-y_{0}\right)}^{2}. \end{array}$$

#### 3.1.3. RSSI Ranging Principle

Through the introduction, this article has some understanding of some methods based on scope and location.  Table 1 compares and summarizes these methods.

In a certain space, there is a certain functional relationship between the signal stop and the distance. The wireless signal has no effect on whether it is reflected or not during the propagation process of the free space, which is a common change, as follows:

(6) $$\begin{array}{c} P_{r}\left(d\right)=P_{t}*G_{t}*G_{r}*{\left(\frac{\lambda }{4\pi d}\right)}^{2}. \end{array}$$

Take the logarithm of formula (6) and transform it into the following:

(7) $$\begin{array}{c} 10\text{lg}\left(P_{r}\left(d\right)\right)=101\text{g}\left(P_{t}*G_{t}*G_{r}*{\left(\frac{\lambda }{4\pi d}\right)}^{2}\right)-20\text{lg}d. \end{array}$$

In formula (7), when measuring distance, the signal strength here can be calculated as follows:

(8) $$\begin{array}{c} 10\text{lg}\left(P_{r}\left(d_{0}\right)\right)=101\text{g}\left(P_{t}*G_{t}*G_{r}*{\left(\frac{\lambda }{4\pi d}\right)}^{2}\right)-20\text{lg}\left(d_{0}\right). \end{array}$$

Next, subtract formula (7) from formula (8) as follows:

(9) $$\begin{array}{c} 10\text{lg}\left(P_{r}\left(d\right)\right)-101\text{g}\left(P_{r}\left(d_{0}\right)\right)=20\text{lg}\left(d_{0}\right)-20\text{lg}\left(d\right). \end{array}$$

Deformation can obtain the following:

(10) $$\begin{array}{c} 10\text{lg}\left(P_{r}\left(d\right)\right)=101\text{g}\left(P_{r}\left(d_{0}\right)\right)+20\text{lg}\left(d_{0}\right)-20\text{lg}\left(d\right). \end{array}$$

It is easy to simplify, and finally, the following is obtained:

(11) $$\begin{array}{c} \text{RSSI}\left(d\right)=A-20\text{lg}\left(d\right). \end{array}$$

**Figure 1:**
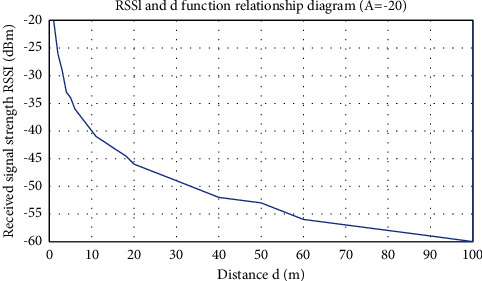
Free-space wireless signal propagation model.

Figure 1 shows the attenuation model curve of the signal model in free space in this case.

Different free-space electrical signal propagation factors are affected differently. After some conditional signals undergo reflection and refraction, the signal strength will be delayed in phase with the free spatial degree. For some received wireless signals, it is not completely measured and transmitted, but during the entire transmission process, it is affected by some environment and can be divided into many components. Finally, because these components are combined, the combined signal usually has a large error with the signal sent by the node under test. Different electrical signals have different principles of propagation in space, and they can all be modeled by signals propagating in free space. The format is as follows:

(12) $$\begin{array}{c} P_{r}\left(d\right)=P_{t}*G_{t}*G_{r}*{\left(\frac{\lambda }{4\pi d}\right)}^{n}. \end{array}$$

After derivation, the principle of nonfree space electric signal propagation model is the same as that in free space. The final model of nonfree space propagation is as follows:

(13) $$\begin{array}{c} \text{RSSI}\left(d\right)=A-10*n\text{lg}\left(d\right). \end{array}$$

**Figure 2:**
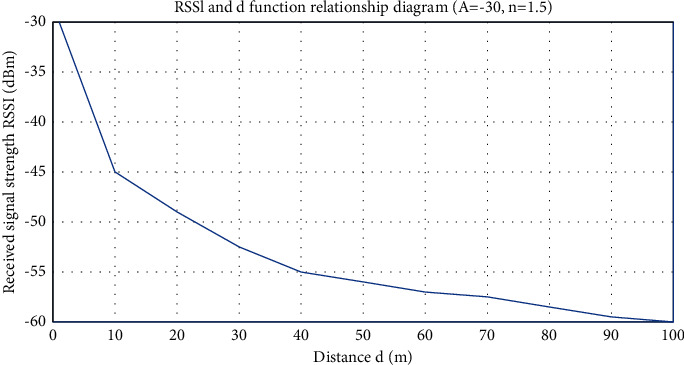
Nonfree space wireless signal propagation model.

To clearly express the model, set *A* = −30, *n* = 1.5, and show the relationship between the signal strength value and the distance in Figure 2.

At present, the nonfree space is very consistent with the actual internal environment. The radio signal propagation model usually adopts the nonfree space model, and the model corresponds to the normal distribution. Based on this condition, the signal strength model is as follows:

(14) $$\begin{array}{c} P_{r}\left(d\right)=P_{0}-n10\text{lg}\left(\frac{d}{d_{0}}\right)+X. \end{array}$$

In the measurement process of the actual RSSI value, the following is mostly used:

(15) $$\begin{array}{c} \text{RSSI}\left(d\right)=A-10*n\text{lg}\left(d\right). \end{array}$$

The relationship between received signal strength and distance, according to the literature, the influence of various environmental factors leads to various attenuation of signal strength.  Table 2 shows that in different environments; the value ranges of A and *n* are also different, because the signal will be affected during the propagation process, so different environments have different values for A and *n*.

In order to further study the influence of A and *n* on the distance propagation model, this paper chooses two different methods for experiments..

**Figure 3:**
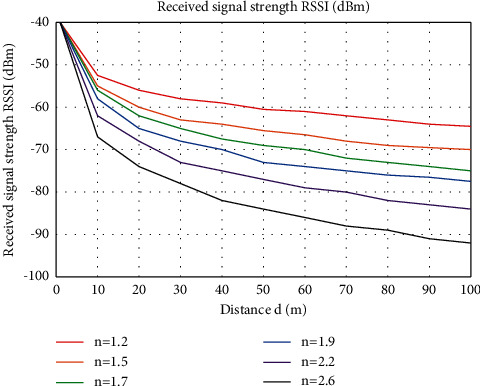
The relationship between distance *d* and signal intensity under different values of *n*.

**Figure 4:**
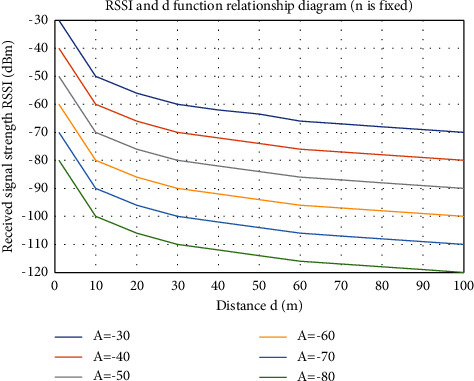
The relationship between distance d and signal strength under different *A* values.

1. Set A to a constant value, obtain various signal attenuation parameters, that is, various values of *n*, and substitute them into the equation to obtain the ratio diagram in Figure 3. *n* increases from top to bottom, and the signal strength changes with time. As the distance increases, the signal strength changes differently, but generally speaking, the larger the value of *n*, the faster the signal strength value decreases.
2. After the correction of the value of *n*, different parameters need to be selected, and the signal strength and distance of different parameters will be affected to a certain extent.  Figure 4 shows several curves under the same influence, different attenuation trends, and different distances affected by different signal strengths.

The following is a process of estimating the values of parameter *A* and parameter *n* using linear regression:

(16) $$\begin{array}{c} p_{i}=-10\text{lg}d_{i},\;i=1,2,3,\ldots ,m. \end{array}$$

Among them, *m* represents the number of times the signal strength is collected, and the following parameter *A* and parameter *n* can be estimated from (16), as shown in the following:

(17) $$\begin{array}{c} n=\hat{n}=\sum_{i=1}^{n}\left(p_{i}-p\right){\text{RSSI}}_{I}l\sum_{i=1}^{n}{\left(p_{i}-p\right)}^{2}, \end{array}$$

(18) $$\begin{array}{c} A=\bar{\bar{Q}}-np. \end{array}$$

Among them, the following is shown:

(19) $$\begin{array}{c} p=\frac{1}{m}\sum_{i=1}^{m}p_{i}, \end{array}$$

(20) $$\begin{array}{c} Q=\frac{1}{n}\sum_{i=1}^{n}\text{RSSI}. \end{array}$$

The effect of this coefficient is used to regress the coefficient, as shown in the following:

(21) $$\begin{array}{c} R^{2}=\frac{\sum_{I=1}^{N}{\left(R_{i}-Q\right)}^{2}}{\sum_{I=1}^{N}{\left({\text{RSSI}}_{i}-\text{RSSI}\right)}^{2}}. \end{array}$$

Among them, the following is shown:

(22) $$\begin{array}{c} R=-\left(A+10n\text{lg}d_{i}\right). \end{array}$$

Under different regression fitting tests, certain experiments need to be measured. The distance between two nodes in different experimental environments is used as the measurement value. The final measurement data is shown in Table 3.

In this article, the generated RSSI value is filtered and preprocessed. Filtering is especially important for obtaining stable values close to the true value of signal strength because it lays the foundation for improving positioning accuracy by obtaining more accurate RSSI values. There are many kinds of filtering methods at present, and the average filtering is the averaging method.  Table 4 shows the corresponding distance of RSSI value before and after filtering and error distance data table.

The following is obtained:

(23) $$\begin{array}{c} f\left(\text{RSSI}\right)=\frac{1}{n}\sum_{i=1}^{n}{\left(\text{RSSI}\right)}_{I}. \end{array}$$

The selection of Gaussian filtering is the most reasonable. The following is obtained:

(24) $$\begin{array}{c} f\left(\text{RSSI}\right)=\frac{1}{\sigma \sqrt{2\pi }}{\text{e}}^{-{\left(\text{RSSI}-\mu \right)}^{2}/2{\text{e}}^{2}}. \end{array}$$

Among them, the following is shown:

(25) $$\begin{array}{c} \mu =\frac{1}{n}\sum_{i=1}^{n}{\left(\text{RSSI}\right)}_{i}, \end{array}$$

(26) $$\begin{array}{c} \sigma =\sqrt{\frac{1}{n}\sum_{i=1}^{n}{\left({\text{RSSI}}_{i}-\mu \right)}^{2}}. \end{array}$$

Regarding how to remove some small probability options, this article deals with some models.

(27) $$\begin{array}{c} \varepsilon \leq \frac{1}{\sigma \sqrt{2\pi }}e^{-{\left(\text{RSSI}-\mu \right)}^{2}/2e^{2}}\leq 1. \end{array}$$

### 3.2. Theoretical Basis of Artificial Intelligence System

The core difference between artificial intelligence and traditional computer technology lies in its powerful learning ability and nonlinear task processing ability. In the face of nonlinear problems, artificial intelligence first integrates and analyzes the low-level information contained therein and then obtains high-level concepts and information through information interpretation and information reasoning. Moreover, when the user is operating the system, artificial intelligence can record and learn the user's habits and tendencies, and when the same user uses it next time, it can actively screen and filter information, thereby improving work efficiency.

The traditional way of computer operation is to issue different instructions and perform different tasks through a source file composed of codes. When the information facing is clear and directed, it can be effectively processed. When the information in front of it has strong vagueness, it is difficult to process. Artificial intelligence works intelligently by simulating humans. In the face of uncertain information, it can provide professional treatment through reasoning and self-study. Moreover, as the scale of processing such information becomes larger and larger, the ability of artificial intelligence to process such information in the process of self-learning has gradually increased.

## 4. Application of Artificial Intelligence System in Enterprise Management

### 4.1. Analysis of Cloud Computing Mode of Enterprise Management

**Figure 5:**
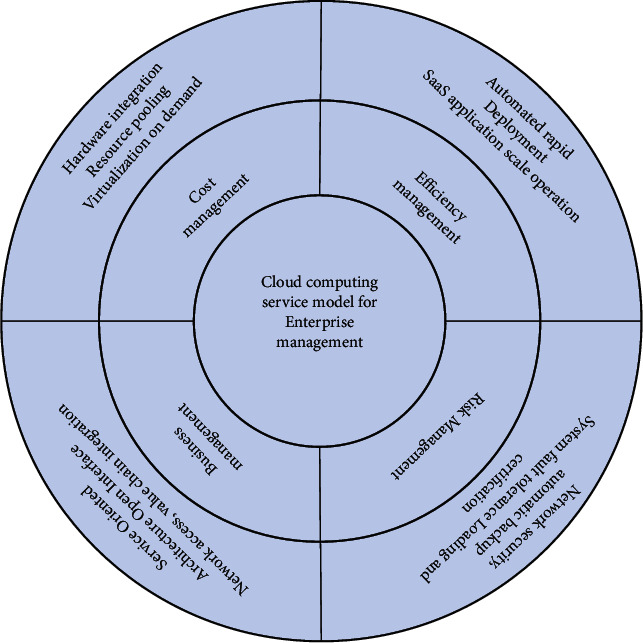
Cloud computing service model for enterprise management.

The manifestation of the powerful capabilities of cloud computing must be combined with the real needs of all aspects of enterprise management, and the close combination of the two can play an important role in enterprise management. This article starts from the four key aspects of enterprise management, cost management, efficiency management, risk management, and business management, and analyzes what cloud computing services should be used to promote the better development of the enterprise, as shown in Figure 5.

#### 4.1.1. The Importance of Enterprise Cost Management

The cost control of an enterprise refers to the need for accounting analysis, decision-making, and management of production and operation costs under different management systems. Different cost management will run through different forecasting decisions, as well as cost planning and analysis issues, cost management, cost estimation, and other functions. The goal is to obtain the greatest benefits at the lowest cost by using scientific and wise management techniques based on product and service quality assurance. Cost control is one of the important parts of business management. Cost control is very important to help companies increase production and costs and improve working conditions. This reflects the overall management level of the company.

Companies can reasonably increase investment in technology, manufacturing, sales, operations, and so on, driven by user needs and satisfaction, and obtain greater profits. This short-term investment can bring long-term benefits. Today, cost control involves many aspects, including not only the connection between production and consumers, but also the use of human resources, equipment, and technological improvements. With the development of Internet technology and the rapid development of rational use of information technology, it has become an important means of controlling enterprise costs.

#### 4.1.2. The Combination of Cloud Computing Technology and Cost Management

*(1) Hardware Integration*. Based on the proposal of semiconductor chips, Intel's founders followed a certain Moore's Law. According to the number of different transistors placed at different costs, the performance can also be improved between different months. However, some studies have shown that due to R&D costs high technology and close to the physical limit, different Moore's Laws require different facilities to complete the use of computer room space and electrical equipment. It is also necessary to pay special attention to the high integration of some storage equipment and switches before they can improve the enterprise. Among the cost management issues.

*(2) Resource Pooling*. In terms of resource pooling, different resource configurations require global resource management and allocation. All IT resource packages are developed based on cloud computing for server storage and other hardware resources. Different application software and application resources will be the virtual pool resources are integrated one by one. Different technical methods require different integrations in terms of efficient management and scheduling. Based on system cloudification, the resource pool needs to perform cloud computing services on the one hand to manage resources more efficiently. On the other hand, to save enterprise hardware development, it is necessary to provide enterprises with a wider range of software and hardware resources, and to integrate and share purchase costs.

*(3) Distribution on Demand*. On-demand distribution is one of the biggest advantages of cloud computing technology. For enterprises, on-demand distribution can be understood as payment based on the final utility they receive. This kind of service provided by cloud computing technology relies on two different main links. Each safe and reliable resource billing system needs to provide support infrastructure on the basis of a cloud computing and use several well-run SaaS services Layer applications interact with various interfaces of the platform to provide payment services. In addition, these services also require appropriate real-time tracking reports to provide enterprises with invoices and payment receipts.

*(4) Virtualization*. Different virtualization designs use cloud computing based on physical resources, making the integrated management of various basic resources more convenient. The application of virtualization technology in enterprise management should mainly solve infrastructure virtualization, virtual local area network, virtual private network, and some other network technologies provided for users. Different storage units are logically accessed for storage virtualization and synthetic abstraction. Logical resources need to be systematically virtualized on the server. Systematic virtualization is a widely used application that can be completed by many operating virtual systems at the same time. If you want to realize software virtualization, enterprise users should use office management, development and other applications can use software virtualization technology, not limited to a single client, but you can use your own applications on different devices, and the data is stored and managed uniformly on the server. In short, virtualization technology is increasing the number of computing, storage, and applications.

#### 4.1.3. The Importance of Corporate Efficiency Management

The improvement of enterprise efficiency is an important part of scientific management. Enterprises should pay attention to improving the efficiency of corporate governance at any stage of development. Under different stages and conditions, adjust management and core technology to adapt to internal and external conditions to achieve maximum efficiency. To improve the efficiency of the organization, the first thing to remember is to formulate complete management regulations and performance appraisal systems to enable employees to perform their duties and clarify goals. The second is that different departments must have different arrangements and reasonable work processes to coordinate. With the popularization of information technology, its role in corporate governance is becoming greater and greater. Different simplifications of corporate work processes are associated with companies investing under specific conditions. This aspect has a great relationship, and investors under certain conditions should be allowed to effectively improve the efficiency of corporate governance. Then, these tasks are performed in the shortest cycle to achieve the set work goals.

In the process of operation and management of some small and medium-sized enterprises, due to imperfect management system, reliable communication mechanism, and low management efficiency. The application of information technology such as OA and ERP may alleviate this problem to a certain extent, but the method is not flexible enough and the effect is not obvious. Like the traditional information management model, companies still rely on manual maintenance and software upgrades. In addition, the one-time purchase of management software is strictly limited, and resources need to be shared. If the resources of colleges and universities cannot be shared, it means that the cooperation between various departments is not tacit, the responsibility system is not clear enough, and the level of relationship between the quality of employees is also different. It is very large, so the efficiency of some business management cannot be improved.

### 4.2. Combination of Efficiency Management and Cloud Computing

#### 4.2.1. Automated Rapid Deployment

The cloud platform infrastructure is created. After the different architecture resource pools are created, the terminal system software needs to be deployed to improve the management efficiency of the enterprise and make cloud computing more flexible and faster, which can save the most time and energy.

Based on the use of appropriate task mobilization systems, cloud computing will use different hardware systems to be compatible with each other and then mobilize services between different environments and systems. The cloud resources and application scales required for enterprise operations vary greatly. Cloud computing is used to attract the dynamic allocation of resources. Due to the relatively high uncertainty and dynamics, some functions such as hot migration of virtual computing nodes and virtual desktop wholesale are required. In addition to the provision of some automated application resources, it is necessary to appropriately increase or decrease the resources used by the system, automatically activate services, and provide sustainable application services. It provides automated strategic management plans according to various priorities such as needs and time. It also has automated statistical analysis functions, which can collect basic event data and summarize it in a database. According to preselected faults, resources, and business statistics, statistics for the enterprise the solution provides a variety of automation support.

#### 4.2.2. SaaS Application

For different enterprises, the value provided by SaaS is more diversified. For some corporate governance, including human resource management, financial management, and so on, SaaS needs to provide more services. In terms of management software model, compared with traditional enterprises, different cloud computing services require unified upgrades and tedious management and maintenance in terms of centralized management. Based on some time-consuming and laborious work, enterprise users will also choose some application software for development according to their own resource needs. This access requirement can improve the efficiency and level of enterprise management. Therefore, cloud computing services for enterprise management need to pay attention to the advantages and characteristics of the SaaS layer, allowing users to apply more stable, secure, and flexible software at a low cost.

#### 4.2.3. Operation of Large Data Centers

Different technology packages require the company's data center to provide some network service leasing. The data center integrates hardware equipment such as large-scale servers, storage and switching equipment, and large-scale clusters significantly reduce corporate costs. While the scale of the management center continues to expand, the complexity of the maintenance center also increases with the increase in costs, and more importantly, it affects the efficiency of enterprise management. Different types of enterprises need to build their own data centers to provide better cloud computing support for the newly constructed green data centers. For different deployments, Tesco's technology uses a load balancing mode on the central management program to implement system integration and data central server upgrade, intelligent power management, and dynamic storage allocation functions. The comprehensive green data center solution based on cloud computing technology supports large-scale data center expansion, which plays a key role in reducing enterprise costs and improving business management.

### 4.3. Application of Cloud Computing Model for Enterprise Management

**Figure 6:**
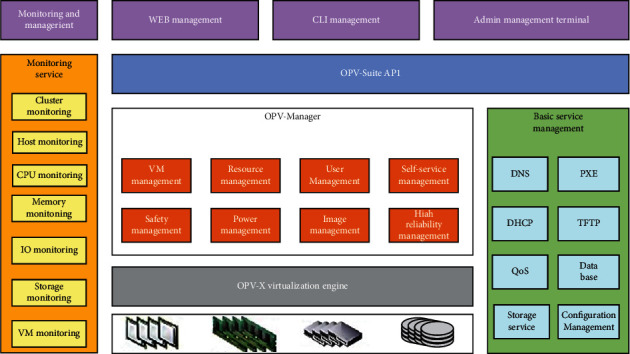
Functional diagram of Hanbo's virtualization management system.

The data center area is an important part of the entire desktop resource maintenance area. Different administrators can allocate according to each user's equipment and storage. All user data is stored in the data center backend center. The data center area is responsible for the integration, management, and backup of user data and the deployment of various security measures. The Opzoon virtualization management system combines intelligent virtual machine management, high scalability, and flexibility without losing control of resources and virtual machines. The main functions are shown in Figure 6.

## 5. Conclusion

In the future, artificial intelligence will play an important role, and one of the important factors is expected to significantly increase the speed of data processing and communication. With the advancement and popularization of 5G technology, artificial intelligence will play an increasingly important role in all aspects of human production and life. With the development of computer technology, artificial intelligence systems have also been applied to computers, allowing artificial intelligence to contribute to the further development of science and technology. Compared with traditional computer technology, the unique characteristics of artificial intelligence systems help to promote the update of computer software and hardware, while maintaining the security and stability of the computer network economically and economically and help improve its efficiency in responding to the new social era and promote the further development of all walks of life. The performance of the artificial intelligence system is very important, and the method of combining multi-dimensional data helps to improve the performance of the artificial intelligence system. Wireless network sensor technology is a subject with wide application potential. As the main component of wireless network sensor nodes, it has high research value. This article proposes a ZigBee wireless sensor network node design scheme based on Arduino. It elaborates on two aspects: hardware system node design and implementation, software system node design and implementation, and node function test. It also tests system performance and checks the feasibility of the design scheme.

## Figures and Tables

**Table 1:** Comparison of several positioning methods based on distance positioning.

Technical name	Features	Power consumption and cost	Positioning accuracy
TDOA	Need ultrasonic auxiliary equipment	High	High
AOA	Need antenna array signal transceiver	High	High
RSSI	Need to accept RSSI value node module	Lower	Higher

**Table 2:** Value ranges of parameters A and *n* in different environments.

Environment	A(dB)	n
Office	(−51.0 to −21.5)	(1.2–2.6)
Stairway	(−58.2 to −29.5)	(1.8–5.7)
Bedroom balcony	(−62.4 to −22.5)	(2.5–3.7)
Hall	(−60.2 to −48.3)	(2.6–3.8)
Laboratory	(−70.5 to −30.5)	(3.9–5.5)
Courtyard	(−71.5 to −38.5)	(3.3–3.9)
Playground	(−70.5 to −48.1)	(4.5–5.4)
Forest	(−80.5 to −25.5)	(1.5–3.8)
Sandy beach	(−86.5 to −56.4)	(2.2–3.8)
Garden	(−88.5 to −40.6)	(3.4–4.5)

**Table 3:** Corresponding data table of signal strength RSSI value and distance.

Distance (m)	RSSI value (dBm)	Distance (m)	RSSI value (dBm)	Distance (m)	RSSI value (dBm)
1	−38	1.8	−32	2	−35
2.5	−37	3	−45	3.5	−45
4	−42	4.5	−40.5	5	−43.5
5.5	−45.5	5	−47.8	6.5	−46.5
7	−48	7	−47.5	8	−47
8.5	−47	9	−44.5	9.5	−48.5

**Table 4:** Corresponding distance of RSSI value before and after filtering and error distance data table.

Actual distance (m)	RSSI corresponding distance before filtering (m)	Error distance before filtering (m)	RSSI corresponding distance after filtering (m)	Error distance after filtering (m)
1	1.25	0.20	1.12	0.18
2	1.35	0.35	2.24	0.22
3	2.62	0.40	2.68	0.32
4	3.52	0.42	3.28	0.24
5	4.32	0.68	4.36	0.36
6	5.27	0.71	5.57	0.49
7	6.25	0.65	6.46	0.61
8	7.01	0.91	7.32	0.68
9	7.80	1.14	8.04	0.96
10	8.79	1.21	8.81	1.21

## Data Availability

The data used to support the findings of this study are available from the author upon request.
